# Complete genome sequence of copper-tolerant *Mammaliicoccus lentus* strain BARC

**DOI:** 10.1128/mra.00885-25

**Published:** 2025-10-17

**Authors:** Alwar Ramanujam Padmavathi, Hiren Joshi, Dhamodharan Prabhu, P. Sriyutha Murthy, Y. V. Nancharaiah

**Affiliations:** 1 Biofouling and Biofilm Processes Section, Water and Steam Chemistry Division, Bhabha Atomic Research Centre 539779 , Kalpakkam, India; 2 Homi Bhabha National Institute, Anushaktinagar 232022, Mumbai, India; 3 Center for Bioinformatics, Karpagam Academy of Higher Education 231537, Coimbatore, Tamil Nadu, India; University of Wisconsin-Madison, Madison, Wisconsin, USA

**Keywords:** antimicrobial resistance, copper resistance, genome annotation, *Staphylococcaceae*, marine biofilm

## Abstract

We report the complete genome sequence of *Mammaliicoccus lentus* strain BARC, a copper-tolerant, biofilm-forming bacterium isolated from marine biofilms formed on titanium coupons exposed to the Bay of Bengal. The genome consisted of a single circular contig of 2.9 Mb with 31.89% GC content, 2,988 genes, including 2,623 coding sequences.

## ANNOUNCEMENT

*Mammaliicoccus lentus,* formerly classified under *Staphylococcus lentus*, is a Gram-positive, catalase-positive, aerobic cocci belonging to the phylum Bacillota and family Staphylococcaceae (1, 2). *M. lentus* is widely recognized as part of the commensal flora in various farm animals, and it has been isolated from humans, food products, and aquatic environments (3).

**Fig 1:**
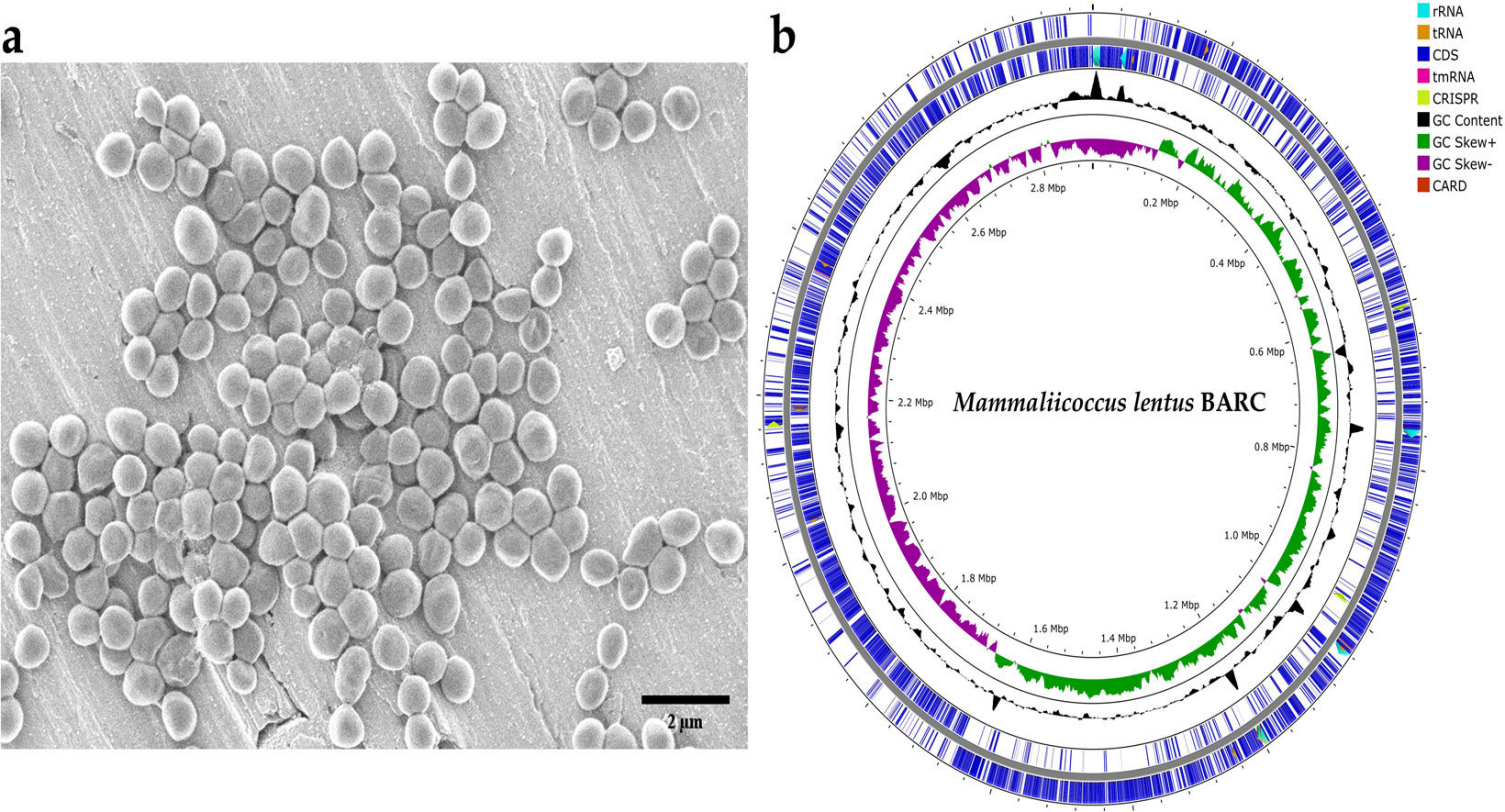
(**a**). Scanning electron microscope image of *Mammaliicoccus lentus* biofilm (24 h) formed on titanium coupon. (**b**). Genome map of *M. lentus* strain BARC.

In our previous study (2018), we reported a prolific biofilm-forming *M. lentus* strain from marine biofilms formed on titanium coupons (Fig. 1a) deployed in the coastal waters at Kalpakkam (12.5238° N, 80.1568° E), South East Coast of India (4). The isolate exhibited substantial copper tolerance and formed confluent biofilms upon exposure to Cu(II) ions (4). Here, we report its complete genome sequence (Fig. 1b). The bacterial isolate was maintained on Zobell marine agar plates by regular streaking. It was cultured in Zobell marine broth at 30°C and 110 rpm for DNA extraction. Genomic DNA was extracted from an overnight grown liquid culture using the QIAamp DNA Mini Kit (Qiagen) and quantified with a Qubit 2.0 fluorometer. The sequencing library was prepared using the Oxford Nanopore ligation sequencing kit, following the manufacturer’s protocol (5). For library preparation, 1 µg of genomic DNA was used without applying any shearing steps. DNA was purified with 0.6× AMPure XP beads to enrich for long fragments prior to the end-preparation. Long fragment buffer (LFB) was used for bead washing before the elution of the adapter-ligated library to selectively enrich >3-kb fragments for sequencing. The prepared library was loaded onto a MinION flow cell for sequencing. Base calling was performed using the Guppy utility integrated into the MinKNOW software (6). The sequencing run generated approximately 129,000 reads with an N50 of 2.9 Mb. Read quality was assessed with Nanoplot v 1.46.1 and FASTQC v 0.12.1 (7, 8), while adaptor trimming was carried out using Porechop v 0.2.4 (9). The trimmed sequences were further *de novo* assembled using Flye v 2.9.6 (10). This resulted in a single circular contig of 2,912,039 bp with 53× coverage and a GC content of 31.89%. The genome was annotated using the NCBI Prokaryotic Genome Annotation Pipeline (PGAP) (11), yielding 2,623 CDSs, 19 rRNAs, 57 tRNAs, and a total of 2,998 genes as shown in Table 1. Seven antimicrobial resistance genes were detected using the comprehensive antibiotic resistance database (CARD) RGI v 6.0.3 in Prokka v 1.14.6 (12, 13). Additionally, CRISPR-CasFinder v 4.2.20 detected two CRISPR-Cas loci (14). Comparative genomic analysis with related strains revealed that *M. lentus* BARC has a slightly larger genome that indicates genomic expansion contributing to robust biofilm formation and enhanced resistance to heavy metals facilitating its survival in dynamic marine environment.

**TABLE 1:** Assembly and genome features of *M. lentus* strain BARC

Description	Values
Genome size	2.9 Mb
GC content %	31.89
Number of contigs	1
Resistance genes	7
CDS	2,623
rRNAs	7, 6, 6 (5S, 16S, 23S)
tRNAs	57
ncRNAs	4
Total number of genes	2,988

## Data Availability

The complete genome sequence of *Mammaliicoccus lentus* strain BARC has been deposited in GenBank under accession number CP195296. Raw sequences were deposited in the Sequence Read Archive (SRA) database under Bioproject accession number PRJNA1282891 and SRA accession number SRP607944.

## References

[1] Becker K, Heilmann C, Peters G. 2014. Coagulase-negative Staphylococci. Clin Microbiol Rev 27:870–926. doi:10.1128/CMR.00109-13 25278577 PMC4187637

[2] Schleifer KH, Geyer U, Kilpper-Bälz R, Devriese LA. 1983. Elevation of Staphylococcus sciuri subsp. lentus (Kloos et al.) to species status: Staphylococcus lentus (Kloos et al.) comb. nov. Syst Appl Microbiol 4:382–387. doi:10.1016/S0723-2020(83)80022-8 23194736

[3] Madhaiyan M, Wirth JS, Saravanan VS. 2020. Phylogenomic analyses of the Staphylococcaceae family suggest the reclassification of five species within the genus Staphylococcus as heterotypic synonyms, the promotion of five subspecies to novel species, the taxonomic reassignment of five Staphylococcus species to Mammaliicoccus gen. nov., and the formal assignment of Nosocomiicoccus to the family Staphylococcaceae. Int J Syst Evol Microbiol 70:5926–5936. doi:10.1099/ijsem.0.004498 33052802

[4] Padmavathi AR, Sriyutha Murthy P, Das A, Nishad PA, Pandian R, Rao TS. 2019. Copper oxide nanoparticles as an effective anti-biofilm agent against a copper tolerant marine bacterium, Staphylococcus lentus. Biofouling 35:1007–1025. doi:10.1080/08927014.2019.1687689 31718302

[5] Quick J, Loman N. 2014. Bacterial whole-genome read data from the Oxford Nanopore Technologies MinION nanopore sequencer. Gigascience 2. doi:10.5524/100102

[6] Wick RR, Judd LM, Holt KE. 2019. Performance of neural network basecalling tools for Oxford Nanopore sequencing. Genome Biol 20:129. doi:10.1186/s13059-019-1727-y 31234903 PMC6591954

[7] De Coster W, D’Hert S, Schultz DT, Cruts M, Van Broeckhoven C. 2018. NanoPack: visualizing and processing long-read sequencing data. Bioinformatics 34:2666–2669. doi:10.1093/bioinformatics/bty149 29547981 PMC6061794

[8] Wingett SW, Andrews S. 2018. FastQ screen: a tool for multi-genome mapping and quality control. F1000Res 7:1338. doi:10.12688/f1000research.15931.2 30254741 PMC6124377

[9] Bonenfant Q, Noé L, Touzet H. 2023. Porechop_ABI: discovering unknown adapters in Oxford Nanopore Technology sequencing reads for downstream trimming. Bioinform Adv 3:vbac085. doi:10.1093/bioadv/vbac085 36698762 PMC9869717

[10] Kolmogorov M, Yuan J, Lin Y, Pevzner PA. 2019. Assembly of long, error-prone reads using repeat graphs. Nat Biotechnol 37:540–546. doi:10.1038/s41587-019-0072-8 30936562

[11] Tatusova T, DiCuccio M, Badretdin A, Chetvernin V, Nawrocki EP, Zaslavsky L, Lomsadze A, Pruitt KD, Borodovsky M, Ostell J. 2016. NCBI prokaryotic genome annotation pipeline. Nucleic Acids Res 44:6614–6624. doi:10.1093/nar/gkw569 27342282 PMC5001611

[12] Alcock BP, Huynh W, Chalil R, Smith KW, Raphenya AR, Wlodarski MA, Edalatmand A, Petkau A, Syed SA, Tsang KK, et al.. 2023. CARD 2023: expanded curation, support for machine learning, and resistome prediction at the Comprehensive Antibiotic Resistance Database. Nucleic Acids Res 51:D690–D699. doi:10.1093/nar/gkac920 36263822 PMC9825576

[13] Seemann T. 2014. Prokka: rapid prokaryotic genome annotation. Bioinformatics 30:2068–2069. doi:10.1093/bioinformatics/btu153 24642063

[14] Couvin D, Bernheim A, Toffano-Nioche C, Touchon M, Michalik J, Néron B, Rocha EPC, Vergnaud G, Gautheret D, Pourcel C. 2018. CRISPRCasFinder, an update of CRISRFinder, includes a portable version, enhanced performance and integrates search for Cas proteins. Nucleic Acids Res 46:W246–W251. doi:10.1093/nar/gky425 29790974 PMC6030898

